# Sustainable Nanotechnology and Artificial Intelligence to Empower Image-Guided Therapy for Precision Healthcare

**DOI:** 10.34133/bmef.0150

**Published:** 2025-07-17

**Authors:** Drishya Prakashan, Ramya PR, Ajeet Kaushik, Sonu Gandhi

**Affiliations:** ^1^ BRIC-National Institute of Animal Biotechnology (NIAB), Hyderabad 500032, Telangana, India.; ^2^ BRIC-Regional Centre for Biotechnology (RCB), Faridabad 121001, Haryana, India.; ^3^ NanoBioTech Laboratory, Department of Environmental Engineering, Florida Polytechnic University, Lakeland, FL, USA.

## Abstract

Nanotechnology has substantially advanced imaging, therapy, and clinical techniques, playing a crucial role in the development of sustainable functional materials in biomedical engineering. Nanoparticles, used as contrast agents in multimodal imaging, offer notable advantages due to their high surface area-to-volume ratio, enabling functionalization with targeting ligands for improved specificity and sensitivity. They can also carry multiple imaging agents or therapeutic drugs, promoting theranostics, an approach combining diagnosis and treatment. However, the need for high-dose contrast agents raises concerns about nanoparticle toxicity. Green nanotechnology addresses this by developing sustainable nanoparticles through eco-friendly synthesis methods, reducing environmental and health risks. Moreover, by using this method, safer imaging agents that align with current health standards can be generated. In parallel, recent advancements in artificial intelligence (AI) are transforming imaging applications. Beyond simple automation of image interpretation, AI is enhancing image acquisition, management, and interpretation, signaling a future where intelligent systems play a key role in healthcare. This review explores the diverse nanomaterials utilized as contrast agents in multimodal imaging, highlights the importance of green nanotechnology in minimizing toxicity, and emphasizes on the important role of AI in imaging and image-guided therapy. Together, these innovations are advancing precision healthcare, promising a future where diagnostics and treatment are not only more effective but also sustainable.

## Introduction

**Fig. 1.**
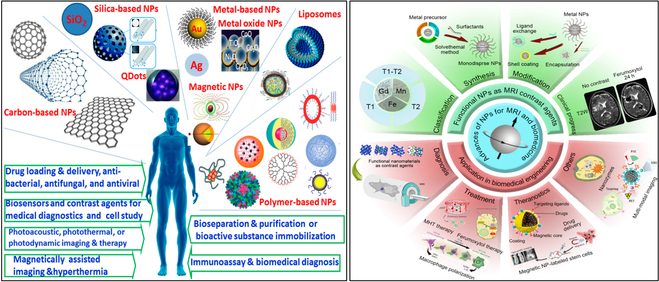
The categorization, creation, and modification of different applications including imaging, diagnostics, drug delivery and tracking, nanoenzyme, and other hotspots (reprinted from [142] an open-access article from MDPI and from [143] with copyright permission obtained from John Wiley and Sons).

The integration of nanotechnology with artificial intelligence (AI) has catalyzed a new era in healthcare, combining the advantages of precise material design with data-driven insights to improve diagnostics, treatment approaches, and personalized medicine. Nanotechnology, a field that uses materials at the atomic and molecular levels, has led the way for the growth of nanomaterials including nanoparticles (NPs), nanoprobes, and nanosensors [1,2]. In particular, these substances were incorporated into biological systems for very selective targeting—to improve the specificity and efficiency in medicine. The particles now have acquired a significant role in the imaging and drug delivery methods, with the capability of very early detection and treatment of diseases (Fig. 1) [3,4].

Additionally, NPs have been modified to function as contrast agents for multimodal imaging, essentially allowing for simultaneous therapeutic interventions, theranostics by virtue of their superior resolution, and sensitivity as compared to traditional methods [5]. Although clinical applications of nanotechnology have improved, increasing concerns about the environmental dosage and long-term biocompatibility of these nanomaterials have arisen. Traditional NPs, made from metals or synthetic compounds, can be hazardous for instance, toxicity, bioaccumulation, and ecological disturbance because they might harm life in ecosystems [6]. In response, the field of green nanotechnology has emerged, focused on the development of sustainable, biocompatible, and biodegradable nanomaterials [7]. These nanomaterials are made from natural sources like plant extracts or biodegradable polymers, engineered to decompose safely inside biological and environmental systems. Green nanotechnology seeks to reduce the ecological damage of nanomaterials while improving or retaining their efficacy as therapeutics. The present pivot to sustainability must tackle the rising global need for green healthcare technologies.

With these advances in nanotechnology, AI in healthcare has witnessed rapid progress, especially in medical imaging, diagnostics, and personalized treatment planning [8–10]. AI algorithms, particularly those based on machine learning (ML) and deep learning (DL), can process large volumes of complex medical data, including genetic data, proteomic profiles, and medical imaging data [11]. AI systems can identify and analyze corresponding patterns within these datasets, which are sometimes too subtle to be detected by the human eye, thus enabling early diagnosis, prognostic prediction, and individual treatment plan development [12,13]. Apart from diagnostics, AI has an equally critical role in optimizing treatment by predicting how patients will respond to specific therapies, allowing any changes to be made to the treatment regimen on a real-time basis. This is particularly important since the combination of AI and nanotechnology will usher in new frontiers in precision medicine aimed at tailoring healthcare to the needs of the patient. AI will take advantage of real-time inputs from medical imaging and any relevant patient biomarkers and thus dynamically update the treatment protocol for optimal patient outcome ranging from personalized to adaptive healthcare strategies [14,15].

Furthermore, the synergy between AI and nanotechnology stretches beyond the boundary of their healthcare applications into wider fields of sustainability and environmental health [16,17]. This review deals with the complex interrelationship between AI and sustainable nanotechnology in the advancement of modern healthcare. It shows how nanomaterials can enhance imaging and therapy while also assessing the contribution of green nanotechnology toward the development of safer, sustainable medical interventions. With the aid of recent studies and researches, the review also examines how the combination of AI with green nanotechnology brings improvements to the field of personalized, eco-friendly medicine. Key topics to be discussed include AI algorithms for the real-time customization of therapy, sustainable NPs for targeted therapy, and the role of AI and green nanotechnology in improving the healthcare environment.

## Imaging Modalities and the Application of NPs in Image-Guided Therapy

Over the past 3 decades, the integration of molecular detection with anatomical imaging, such as optical imaging, positron emission tomography (PET)/magnetic resonance imaging (MRI), PET/computed tomography (CT), and single-photon emission computed tomography (SPECT) scanners, has advanced clinical diagnostics. Among them, optical imaging is one widely used and cost-effective technology, as it is a readily available and adaptable source of imaging radiation. CT is another type of imaging founded on the utilization of x-ray scanning, tissue attenuation, and computer image reconstruction for the collection of morphological and vascular information pertaining to internal organs. MRI is one of the main noninvasive medical diagnostic methods that rely on the process of exciting nuclei and relaxing magnetic spins for imaging. Another widely used imaging method is radionuclide imaging, which relies on the use of radioactive nuclei for picture reconstruction and detection. PET and SPECT are 2 of the most extensively utilized radioactive imaging modalities. Different imaging methods are available in the biomedical field; however, challenges persist, including the need for standardization and optimization of multiple imaging techniques [18]. The primary goal of multimodal imaging is to balance the benefits and drawbacks of various techniques, particularly sensitivity and resolution, to benefit disease diagnosis, patient layer, therapy response assessment, and drug development. NPs are especially useful for these applications. No single imaging modality can offer all the necessary data, highlighting the importance of multimodal imaging for comprehensive characterization of administered agents and guiding clinical and preclinical decisions. Nanomaterials are frequently used in biomedical domains because of their great physicochemical qualities, strong biocompatibility, ease of surface modification, and simplicity in controlling size and morphology.

Nanomaterials for different imaging techniques, including MRI, PET, x-ray, and SPECT, have a variety of applications since they can target specific areas in vivo and more simply identify disease-related specific biomarkers at both the molecular and cellular levels than standard contrast agents [19,20]. Nanomaterials used for multimodal imaging fall into several classes according to their composition, structure, and properties. These classes encompass a diverse range of materials, each tailored to serve specific functions in various imaging modalities [21]. Various categories of nanomaterials, utilized for multimodal imaging, are metals [silver (Ag), gold (Au), and iron oxide NPs (IONPs)], semiconductor nanocrystals [quantum dots (QDs) and carbon-based nanomaterials inclusive of graphene, carbon, and silica-based NPs], and organic nanostructures (dendrimers and polymeric systems) [22,23]. The selection of nanomaterial varies with the specific prerequisite of the imaging application, including the desired imaging modalities, biocompatibility, and the potential to target specific tissues or cells.

### Metal NPs

#### Iron oxide NPs

**Fig. 2.**
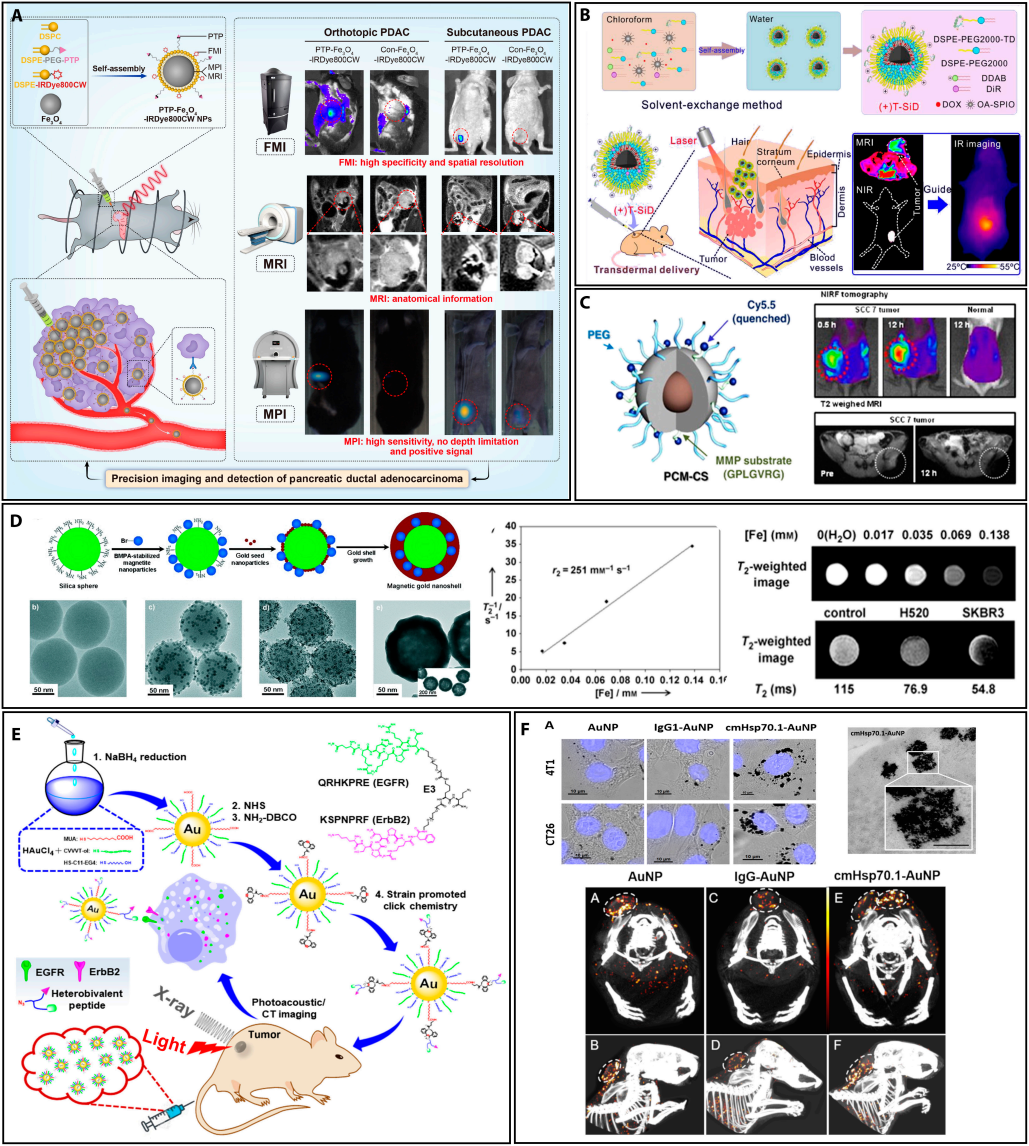
(A) The multimodal imaging of orthotopic and subcutaneous PDAC using a targeted PTP-Fe_3_O_4_-IRDye800CW imaging agent (reprinted from [39] with copyright permission for figure obtained from Elsevier). Multimodality imaging comprises FMI, MPI, and MRI. (B) Iron oxide-based nanoplatform for transdermal delivery. The synthesis process of the superparamagnetic NPs by solvent-exchange method and transdermal administration of (+)T-SiDs for multimodal MR/NIR imaging-guided chemo-photothermal treatment (reprinted from [40] with copyright permission obtained from Elsevier). (C) Iron oxide-based core-shell NPs with 2 different modalities: Magnetic resonance and near-infrared fluorescence guided (NIRG) optical imaging modalities effectively visualized the tumor sections through tail vein injection (reprinted from [41] with copyright permission for figure obtained from Elsevier). (D) The synthesis process of the Mag-GNS and the transmission electron microscopy (TEM) images of amino-modified silica spheres, silica spheres with surface-immobilized Fe_3_O_4_ (magnetite) NPs, silica spheres with Fe_3_O_4_ and AuNPs, and the Mag-GNS. Plot of the spin–spin relaxation rate (T2^−1^) and T2-weighted MR images (reprinted from [43] with permission obtained from John Wiley and Sons). (E) The heterobivalent (HB) peptide specific for EGFR and ErbB2 (HB-AuNPs), which were created for dual photoacoustic/CT imaging, is displayed on the thin layer-protected AuNPs (reprinted from [44] that is an open access article circulated under the Creative Commons Attribution License). (F) Uptake of AuNPs in tumor cells visualized by brightfield, TEM, and tumor detection using spectral CT (reprinted from MDPI [45], which is an open-access article).

One type of metal NPs is IONPs, which is of great interest in the area of multimodal imaging. These NPs, composed of iron oxide, commonly maghemite (γ-Fe_2_O_3_) or magnetite (Fe_3_O_4_), exhibit unique magnetic properties that make them particularly suitable for different imaging modalities [24,25]. IONPs can be utilized to scan tissues or cells that either collect or accumulate, including liver [26], spleen, lymph nodes [27,28], bone marrow, and mononuclear phagocytic organization, where these tissues or cells are tumorigenic or not [7,24]. Additionally, IONP imaging applications include the detection of apoptosis [29], inflammation [30], angiography [31], ruptured atherosclerotic plaque, multiple sclerosis [32], the integrity of the blood–brain barrier [33], and vasculature, including coronary arteries. MRI-optical dual-modal imaging is one of the straightforward multimodal imaging techniques [34]. The presence of IONPs can be detected indirectly by quantifying tissue resistance under a high-intensity pulsed magnetic field during IONP motion [35]. This approach can provide anatomical information and estimate IONP quantity, or enhance resolution in photo-acoustic imaging. It is also useful for dynamic imaging of moving biological systems and can be combined with other imaging techniques [36]. One of the studies demonstrated that AgIONPs can be utilized for precise photothermal therapy and thrombosis imaging, where IONPs functioned as seeding agents to generate spiky AgNPs exhibiting pronounced near-infrared (NIR) absorbance. The AgIONPs were biofunctionalized to enable precise thrombus targeting. The application of photoacoustic and fluorescence imaging techniques substantiated the specific binding of the NPs to thrombi, particularly facilitated by a single-chain antibody designed for activated platelet recognition. In the in vivo experiments, photothermal therapy utilizing AgIONPs reinstated blood flow exclusively in the targeted group, whereas no such restoration was observed in the nontargeted group [37]. Similarly, Deh et al. [38] discovered the aid of superparamagnetic iron oxide (SPIO) NPs for cancer therapy through magnetic fluid hyperthermia (MFH) under an alternating magnetic field (AMF). The study developed a robust quantitative susceptibility mapping (QSM) method for live mice, successfully detecting ferumoxytol deposition in the liver and near tumor peripheries. A combination of magnetic particle imaging (MPI), fluorescence molecular imaging (FMI), and MRI allowed accurate identification of pancreatic ductal adenocarcinoma (PDAC) xenografts (Fig. 2A) [39].

Additionally, a platform that combined superparamagnetic IONPs (SPIONs) for MRI contrast and photothermal therapy, a transdermal enhanced peptide (TD), and cationic lipids to facilitate penetration into skin, and included NIR dye (DiR) and doxorubicin (DOX) for improved chemo-photothermal therapy and NIR imaging was developed. It was observed that the platform could efficiently eliminate tumor with minimal systemic toxicity, demonstrating significant potential for clinical use (Fig. 2B) [40]. Additionally, the conjugation of IONPs with other polymer materials enhanced the imaging capability. An enhanced imaging strategy was developed for the detection of tumors with the aid of Cy5.5-MMP (Cy5.5-Gly-Pro-Leu-Gly-Val-Arg-Gly) substrate and PEG combined IONPs with a thin coating of silica (PCM-CS) and “activatable” dual imaging probe (Fig. 2C) [41]. However, the challenges include improving the properties of IONPs and imaging methods, optimizing IONP concentration, developing a unified imaging approach, creating integrated devices, aligning with specific medical needs, and leveraging IONP compatibility for multimodal imaging. Meeting these challenges will enhance medical imaging for more effective diagnosis and treatment.

#### Gold NPs

In the family of NPs, AuNPs are a subtype that has been the subject of extensive research [42]. These are broadly utilized in the area of medical imaging because of their distinctive optical properties, ease of functionalization, biocompatibility, and ability to contribute to improved contrast and sensitivity. AuNPs, in particular, can be utilized to initiate the controlled release of pharmaceutical medicines and as photothermal therapeutic agents. Magnetic gold nanoshells (Mag-GNS) were suggested by the Hyeon group as a new strategy for MRI imaging and photothermal treatment of breast tumors (Fig. 2D) [43]. Further, ultra-small AuNP with a protective layer was used for cancer detection via photoacoustic and CT imaging. These NPs, called HB-AuNPs, are targeted to cancer cells overexpressing epidermal growth factor receptor (EGFR) and ErbB2 (Fig. 2E). In vivo evaluation of these particles showed the selective uptake by these cancer cells, with peak uptake at 8 h and clearance by 48 h [44]. Anti-plasma membrane heat shock protein 70 (Hsp70) antibodies were used to functionalize AuNPs for tumor-specific multimodal imaging (cmHsp70.1-AuNPs). It was noted that targeting mHsp70 with cmHsp70.1-AuNPs facilitated efficient enhancement and circulation within tumor cells. This targeted approach enabled the application of diverse imaging systems and spectral CT-based tumor delineation in vivo (Fig. 2F) [45].

A noninvasive MR/CT/NIR fluorescence (NIRF) trimodal contrast agent made up of Au and gadolinium coupled to prostate-specific membrane ligand 1 (PSMA1) (AG-Gd@PSMA1 NPs, AGGP) was developed to detect and quantify the prostate-specific membrane antigen (PSMA) expression in prostate cancer (PCa) lesions. AGGP, combined with a high-affinity PSMA ligand (PSMA1), overcomes limitations like nonspecific redistribution and inadequate perfusion of current contrasting agents. It provides multimodal signal enhancement, specific PSMA targeting, myocardial perfusion imaging (MPS) avoidance, and renal-clearable behavior in living mice. The agent exhibits high biocompatibility and safety, suggesting potential for recovering early-stage PCa detection and medical use of multifunctional nanotherapeutics. AuNPs continue to be a subject of intense research in the growth of advanced imaging agents, and their versatility makes them valuable tools in the molecular imaging field [46].

### Semiconductor nanocrystals

#### Carbon-based NPs

**Fig. 3.**
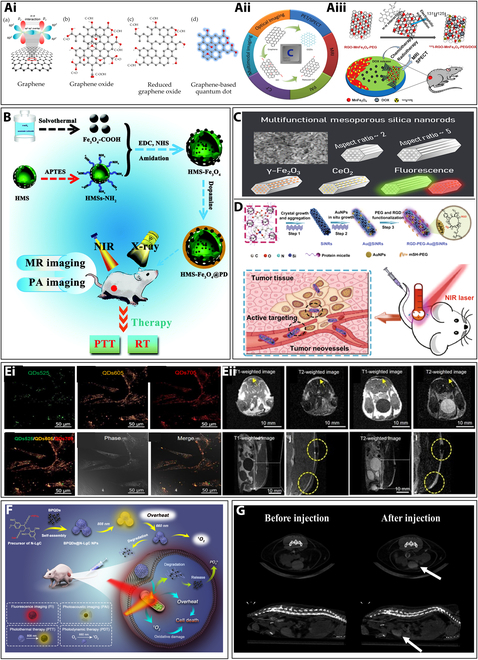
(A) (i) Different graphene-derived nanomaterials, including graphene, GO, rGO, and GQDs (reprinted from [144], which is an MDPI open access article). (ii) Application of graphene nanomaterials for biomedical imaging. This encompasses optical imaging techniques such as fluorescence (FL), 2-photon FL, and Raman imaging, as well as PET/SPECT, MRI, PAI, CT, and multimodal imaging (reprinted from [145] with copyright permission for figure obtained from Elsevier). (iii) Design of nano-graphene-based nanocomposites with multifunctionality for guiding combined radioisotope therapy and chemotherapy in multimodal imaging (reprinted from [49] with copyright permission for figure obtained from Elsevier). (B) Synthesis process of hollow mesoporous silica-coated IONPs (HMS-Fe_3_O_4_@PD NPs) designed for utilization in multimodal cancer theranostics [reprinted from [61] with copyright (2019) Royal Society of Chemistry]. (C) Magnetic mesoporous silica nanorods incorporated with iron and ceria and fluorophores for imaging [reprinted from [62], copyright (2022) American Chemical Society]. (D) Nanotheranostic agents utilizing fluorescent silicon nanorods in multimodal imaging during photothermal therapy (reprinted from [63], which is an open access article). (E) (i) Confocal microscopy images of ASCs labeled with DLU2-NPs. (ii) In vivo MR pictures of labeled ASCs and the transversal pictures of nonlabeled ASCs (reprinted from [65], an open access article from MDPI). (F) BPQDs@N-LgC NPs with a spherical structure for targeted imaging of mitochondria and combined antitumor treatment. The process of modifying lignin and assembling BPQDs@N-LgC NPs, and the versatile use of BPQDs@N-LgC NPs for systemic delivery as a theranostic system, enabling multifunctional therapy and multimodal imaging (reprinted from [66] with copyright permission for figure obtained from Elsevier). (G) In vivo CT of axial sagittal and coronal images of a mouse before injection and after intravenous tail vein injection of CQDs (reprinted from [67] with copyright permission for figure obtained from John Wiley and Sons).

Carbon-based nanomaterials have garnered substantial attention in the imaging domain, attributable to their distinctive characteristics. Their remarkable mechanical, thermal, and optical properties position them as promising candidates for the subsequent generation of theranostic probes. One of the carbon-based materials that have been widely used for imaging is graphene. Graphene and graphene-related nanomaterials stand out as the most widely researched materials due to their numerous exceptional physicochemical properties [5]. It is the strongest, thinnest, and stiffest materials that possess optical features, such as the capability to quench fluorescence, a substantial specific planar surface area, and unparalleled thermal conductivity. Graphene family nanomaterials (GFNs) include graphene, graphene quantum dots (GQDs), graphene oxide (GO), and reduced graphene oxide (rGO) (Fig. 3Ai), which exhibit many exclusive and attractive properties because of structure and geometry [47,48]. Hence, these NPs are widely used in different imaging techniques (Fig. 3Aii).

Biocompatible reduced graphene oxide-manganese ferrite (RGO-MnFe_2_O_4_) nanocomposites demonstrated excellent MRI in mice with 4T1 tumors after intravenous injection. SPECT imaging of ^125^I-labeled RGO-MnFe_2_O_4_-PEG showed a significant accumulation of tumor, emphasizing their potential for use in a range of biological applications (Fig. 3Aiii) [49]. The ability to modify the optical properties of graphene allows for efficient fluorescence quenching and enhancement, enabling precise control in fluorescence imaging applications [50]. The integration of graphene nanomaterials in multimodal imaging holds great promise; however, more research is required to understand and develop graphene-based platforms for multimodal imaging. Carbon nanotubes (CNTs) are another class under the carbon-based nanomaterials that have been explored for various imaging modalities, including photoacoustic imaging, NIR fluorescence imaging, and MRI [51]. Their strong absorbance in the NIR region makes them suitable for deep-tissue imaging [52]. Sobhani et al. [53] demonstrated that oxidized CNT-PEG showed a reduced level of toxicity compared to pure multiwall nanotubes. In photothermal therapy (PTT), O-CNT-PEG significantly reduced melanoma tumor size in mice exposed to a continuous-wave NIR laser diode, thereby depicting its capability as a promising candidate for PTT-based solid tumor elimination. Behnam et al. [54] investigated CNT/AgNP particles for inhibiting B16/F10 melanoma tumors in mice. Evaluating tumor sizes showed enhanced plasmon photothermal therapy efficacy, attributed to increased optical absorption in CNT/Ag compared to CNTs and Ag nanorods alone.

In addition to PTT, CNTs can serve as contrast agents in other modalities such as MRI and CT. Cobalt core/carbon shell NPs (referred to as Cobalt at carbon NPs) were designed to possess dual-modality functionality for both MRI and photoacoustic imaging (PAI), owing to their inherent magnetization and light absorption properties. Upon intravenous injection into glioblastoma-bearing mice, Cobalt at carbon NPs accumulated and retained in tumors. This multifunctional probe enables rapid lesion screening via MRI and provides high-resolution structural morphology and quantifiable tumor data through PAI, making it a capable molecule for dual-modality tumor imaging [55]. Overall, CNTs represent an important platform for multimodal imaging, offering a synergistic approach to obtaining diverse and valuable information from different imaging modalities.

#### Silica NPs

Silica NPs are nanoscale-sized particles composed primarily of silica, a compound made from silicon and oxygen. Silica NPs have been used in the field of various imaging methods including fluorescence imaging, CT imaging, MRI, photoacoustic imaging, and ultrasound (US) imaging [56]. Due to attributes including high colloidal stability, tunable particle size, exceptional biocompatibility, low toxicity, and transparency to light and magnetism, silica NPs manifest significant promise in biomedical applications [57]. Notably, silica NPs exhibit a lack of light absorption and do not interfere with magnetic fields, enabling the preservation of the original optical and/or magnetic properties of incorporated components. Moreover, silica, with its abundance of hydroxyl groups, allows for versatile chemical modifications suitable for loading antibodies and drugs [58]. Considering these inherent characteristics, silica NPs are widely used as the carrier in our imaging system [59].

In a recent study, perfluorohexane (PFH)-encapsulated mesoporous silica nanoparticles (MSNs) combined with indocyanine green (ICG)–polydopamine (PDA) layer and poly (ethylene glycol)–folic acid layering were designed (MSN-PFH@PDA-ICG-PEG-FA) for combined tumor US and NIRF imaging, and also PTT and PDT (photodynamic therapy). The nanocarrier demonstrated efficient ICG loading, enhanced ICG photostability, and improved cellular uptake. Further, in vivo studies showed significant tumor growth inhibition and a 60% healing rate [60]. Similarly, magnetic MSNs coated with PDA were designed by encapsulating ultrasmall IONPs within the hollow mesoporous silica particles and subsequently applying a PDA coating to the particle surface. This engineered nanoplatform exhibited enhanced colloidal stability, improved r1 relaxivity, and a NIR absorption feature, making it suitable for applications in multimodal cancer theranostics (Fig. 3B) [61]. Further, mesoporous silica rods loaded with a cerium compound and surface-functionalized with fluorophores, specifically fluorescamine and Cyanine_5_, facilitated multiple imaging modalities. In vitro biocompatibility assessment in a zebrafish liver cell line (ZFL) showed no cytotoxic effect for dilutions up to 50 μg/ml, which suggested the use of these particles in clinical imaging and therapy (Fig. 3C) [62].

In another study, fluorescent silicon nanorods adorned with AuNPs (Au@SiNRs) were used for targeted multimodal imaging-guided photothermal therapy in tumor applications. Au@SiNRs demonstrated prominent qualities, such as high photothermal conversion efficacy (~43.9%) and sustained performance throughout 5 cycles of NIR laser irradiation. They enable effective simultaneous photoacoustic and infrared thermal imaging. Modified with targeting peptide ligands, Au@SiNRs show improved tumor buildup (~8.74% ID g^−1^). With these benefits, Au@SiNRs precisely ablate tumors in vivo under multimodal imaging guidance (Fig. 3D). Importantly, all mice medicated with Au@SiNRs survived, and no reappearance of the tumor was observed in the 60-d study [63].

#### Quantum dots

QDs represent a versatile class of optical imaging agents amenable to facile stabilization through surface modifications, incorporation of targeted ligands, and utilization as bimodal imaging probes for efficient visualization of tumor angiogenesis. Mulder et al. [64] illustrated the synthesis of water-soluble, paramagnetic, PEGylated, cyclic arginine-glycine-aspartic acid (RGD)-functionalized QDs, serving as a molecular imaging probe applicable for both MRI and fluorescence microscopy. Further, researchers also focused on the development of nanohybrid particles, called DLU2-NPs, which consist of dendron-baring lipids (DLU2 molecules), QDs, and magnetic NPs that can address the current inadequacy of in vivo imaging for the detection of transplanted stem cells. These particles can be utilized to label and image transplanted stem cells. Labeling adipose tissue-derived stem cells (ASCs) with DLU2-NPs did not affect their viability or proliferation. Upon transplantation into mice, the labeled ASCs could be efficiently visualized using in vivo fluorescence and MRI [65] (Fig. 3Ei and Eii).

In a recent study, lignin loaded with chlorin e6 and black phosphorus quantum dots (BPQDs) was used to create BPQDs@N-LgC NPs (Fig. 3F). These NPs were utilized for mitochondria-targeted photothermal and photodynamic therapy, guided by photoacoustic and photoluminescence imaging. When irradiated at 808 nm, the BPQDs generated heat for PAI and tumor growth inhibition. At 660 nm, the BPQDs produced fluorescence and reactive oxygen species (ROS) for further tumor growth inhibition.

Cleavage of the lignin’s β-O-4 bond by photo-triggered ROS enabled the rapid excretion of therapeutic nanomaterials. This approach demonstrated effective healing efficacy in both in vitro and in vivo experiments [66]. Similarly, carbon quantum dots (CQDs) doped with nitrogen and lanthanides (Gd and Yb) were synthesized for improved multimodal imaging. These doped-CQDs provided intense fluorescence and excellent MR and CT contrast properties without any toxicity. Further, in in vivo studies, after intravenous injection, significant MR and CT contrast enhancement was observed in a mouse’s bladder and kidneys [67] (Fig. 3G). Although quantum dots are widely used for imaging, more research should focus on improving their biocompatibility, developing novel surface coatings, and expanding their applications in advanced imaging technologies.

### Organic NPs

#### Dendrimers

Dendrimer-based contrast agents have significant attention due to their well-defined architecture, highly branched interior, and numerous surface functional groups [68]. These distinctive structural characteristics not only make dendrimers efficient for labeling with various radionuclides but also allow for the convenient creation of multifunctional nanomaterials [69]. These nanomaterials are useful for a variety of nuclear medicine applications, including dual or multimodality imaging and theranostics [70,71]. A nanoprobe designed for continuous and tracking T cells utilized poly(amidoamine) (PAMAM) dendrimer-entrapped AuNPs (AuDENPs) conjugated with Fluo-4 for dual-mode CT and fluorescence imaging. Generation 5 (G5) PAMAM dendrimers underwent modification with hydroxyl-terminated polyethylene glycol (PEG) for the encapsulation of 2.0-nm AuNPs. The resulting functional {(Au^0^)25-G5.NHAc-(PEG)14-(Fluo-4)2} nanoprobe facilitated the fluorescent sensing of activated T cells and enabled both CT and fluorescence imaging of injected T cells in vivo. This dendrimer-based nanosystems showed good potential for evolving T cell-based immunotherapy in clinical applications [72].

**Fig. 4.**
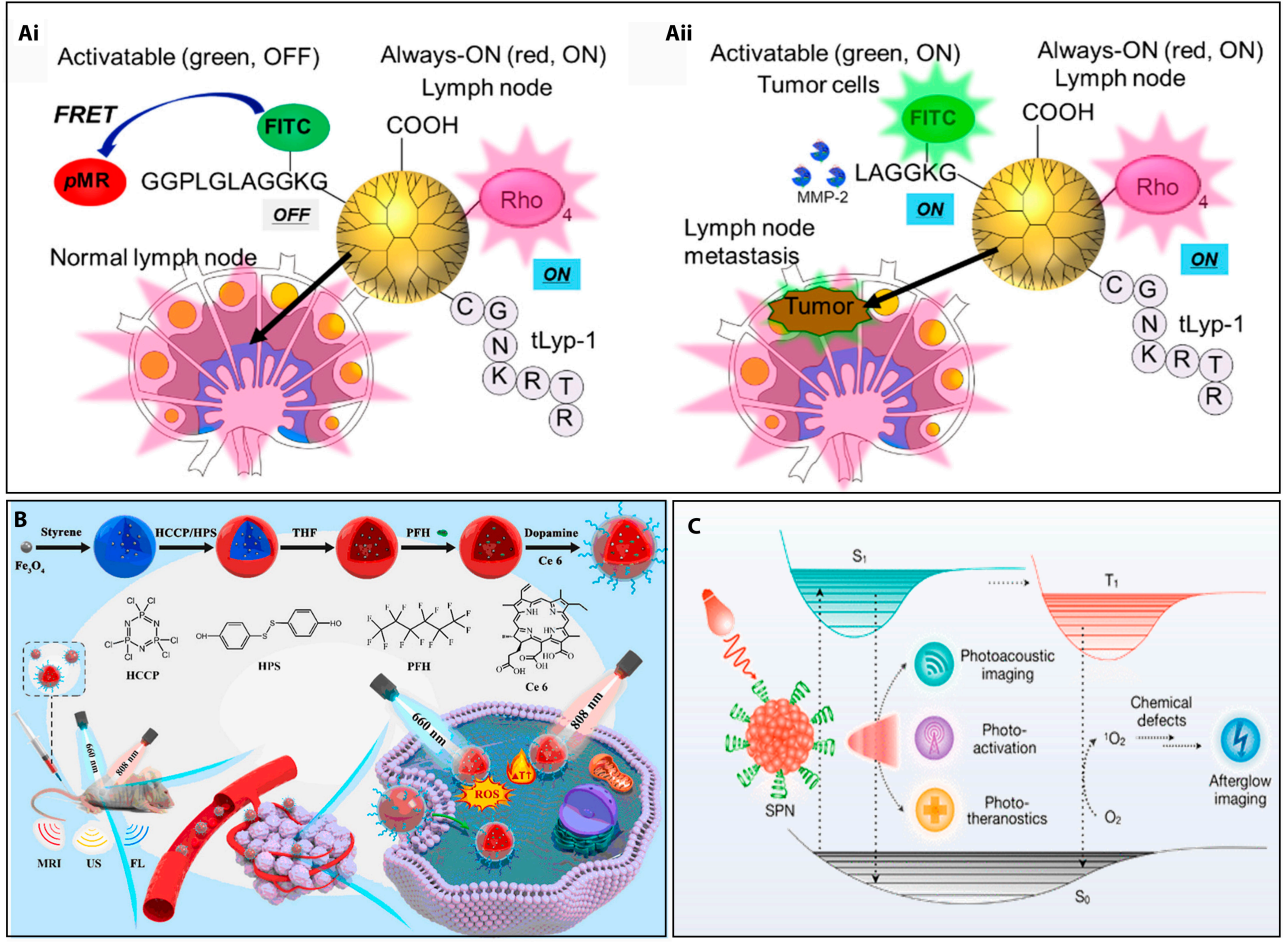
(A) MMP-2 substrate peptide with a quencher (pMR) and a fluorophore [fluorescein isothiocyanate (FITC)] at both ends as an activatable fluorescence probe, combined with a tumor-homing peptide (tLyp-1) to create a dual-color fluorescent carboxyl-terminal dendrimer. Ro (rhodamine B) functions as an invariant probe. Single- and dual-color fluorescent signals from the lymph node containing (i) the normal lymph node and (ii) metastatic tumor cells (reprinted from [73], which is an MDPI open access article). (B) Fe_3_O_4_ NPs and PFH were encapsulated in biocompatible PPZ, allowing for the realization of PTT/enhanced PDT combo therapy guided by US/MR/fluorescence imaging (reprinted from [80] with copyright permission for figure obtained from Elsevier). (C) SPNs’ distinct photophysical characteristics have made deep-tissue molecular imaging incredibly sensitive. Strong charge-transfer backbones in NIR-absorbing SPNs enable the conversion of photoenergy into mechanical acoustic vibrations, enabling photoacoustic imaging that can penetrate tissue to a depth of 1 cm while avoiding the problem of light scattering. Schematic illustration of SPNs for advanced biophotonic applications such as PTT and PDT [reprinted with permission from [82] with copyright (2018) Royal Society of Chemistry].

A fluorescence imaging probe was engineered to facilitate concurrent diagnosis of lymph nodes and tumor cells by employing always-ON and activatable probes with distinct colors. The dendrimer-based probe, integrating a matrix metalloproteinase-2 (MMP-2)-responsive green fluorescence probe, exhibited heightened signals specifically from tumor cells. This strategy holds promise for noninvasive imaging in the detection of cancer metastasis in lymph nodes (Fig. 4Ai and Aii) [73]. For in vivo specialized diagnostic applications, numerous contrast agents based on dendrimers have been appropriately developed, demonstrating significant flexibility for the molecular imaging of organs and other target-specific areas [74]. Small changes in dendrimer size, even those on the nanoscale, have a significant impact on biodistribution, pharmacokinetics, and excretion. To facilitate target-specific imaging, these alterations allow the dendrimer-based contrast agents to localize specifically to regions or organs of attention and assume excretion paths that do not obstruct intended uses. Additionally, the abundance of attachment sites on dendrimers has stimulated the development of novel synthetic chemical methods for multimodality imaging agents [75,76].

#### Polymeric nanomaterials

Polymeric nanomaterials have gained importance in the area of imaging because of their versatility, biocompatibility, and tunable properties. These materials, composed of synthetic or natural polymers, can be engineered to carry imaging agents and exhibit characteristics suitable for various imaging modalities [77,78]. Their flexibility allows for the modification of structures, yielding materials with diverse compositions, sizes, morphologies, and surface properties [79]. Fe_3_O_4_ NPs and PFH encapsulated in biocompatible polyphosphazenes (PPZ) and PDAs guided by US/MR/fluorescence imaging significantly inhibited tumor growth, showing promise for imaging-guided phototherapy (Fig. 4B) [80].

Nanocarriers are known for improved clinical translation of nanomedicine. Through microfluidics, a hybrid lipid–polymer nanocarrier was created, employing hydrodynamic flow focusing to guide the synthesis of lipid–polymer NPs (LiPoNs). These NPs concomitantly encapsulated gadolinium-diethylenetriamine pentaacetic acid (Gd-DTPA) along with irinotecan/Atto 633 compounds for applications in both MRI and optical imaging. Preliminary findings additionally suggested improved drug delivery and enhanced therapeutic efficacy against cancer cells [81].

In the field of advanced biophotonics, there has been progress in creating semiconducting polymer nanoparticles (SPNs) that serve as multimodal light converters, particularly in the NIR range. SPNs exhibit unique photophysical properties, enabling ultrasensitive deep-tissue molecular imaging. NIR-absorbing SPNs with charge-transfer backbones facilitate photoacoustic imaging, overcoming light scattering for centimeter tissue penetration. Phenylenevinylene-containing SPNs store photon energy, emitting long-NIR afterglow with minimal tissue autofluorescence, resulting in ultrahigh signal-to-background ratios. These SPNs have been utilized for molecular imaging of diseases, biomarkers, and physiological indexes in various preclinical animal models (Fig. 4C) [82].

Moreover, new materials as well as combining polymers with well-studied NPs could efficiently improve the imaging. Bimodal sub-5-nm SPIO-5 coated with PEG with attachment of NIR-emitting dye using organosilane coating enabled tracking of their in vivo biodistribution and elimination through various imaging techniques, including high-field MRI, optical, and optoacoustic imaging. The study found that the thickness of the coating has a substantial impact on the pharmacokinetic parameters of the injected SPIO-5, which is interesting [83]. Polymeric nanomaterials provide a building block for the advancement of targeted and multifunctional imaging agents with reduced toxicity and improved biocompatibility. However, research should focus on new polymer formulations and functionalities to advance imaging capabilities and meet the demands of personalized medicine. Table describes some examples of NPs that have been used for different image-guided therapies.

**Table.** Different nanoparticles and their dose/exposure time and principle of action in multimodal imaging

Nanoparticle	Dose/exposure time	Principle of action/application	Performance	Reference
AgIONPs	2–4 mg kg^−1^	Superparamagnetism for photothermal therapy	Efficient photothermal agent to induce hyperthermia and thrombolysis	[37]
Superparamagnetic iron oxide (SPIO)	125–750 μg	Superparamagnetism for MRI quantitative susceptibility mapping for hypothermia	Magnetic hyperthermia for cancer therapy-magnetic nanoparticles heated by an alternating magnetic field to target tumor tissues	[38]
PEGylated Cy7-SPIONs	10 mg Fe/kg, <11 min	Multispectral optoacoustic tomography (MSOT)	Drug delivery, tumor targeting (rapid clearance)	[149]
PTP-Fe_3_O_4_-IRDye800CW nanoparticles	0.01625–0.25 mg/ml	Magnetic particle imaging (MPI) combined with FMI and MRI	Targeting plectin-1 expressed orthotopic pancreatic ductal adenocarcinoma in mice (higher specificity, even distribution, and longer retention effects)	[39]
(+)T-SiDs	4.0 mg/kg for 10 min	MR/NIR imaging-guided synergistic chemo-photothermal therapy	Tumor inhibition and higher biocompatibility in mice cancer models	[40]
Heterobivalent (HB)-AuNPs	2.5 mg/ml	Photoacoustic imaging and CT imaging	Early detection and staging of cancer (good stability, biocompatibility with fast clearance, and contrast-enhancing capability)	[44]
CGNP clusters-RGD	5 mg/ml	Photoacoustic microscopy (PAM) and optical coherence tomography (OCT)	Biocompatibility and photostability	[150]
Graphene quantum dots	4 mg kg^−1^ /10min	Photodynamic therapy	Cancer treatment (^1^O_2_ quantum yield, water dispersibility, photo- and pH-stability, and biocompatibility)	[151]
BPQDs@N-LgC NPs	400 μg/ml/ 10 min	Mitochondria-targeted fluorescence/photoacoustic-guided PTT and PDT	Mitochondria-targeted tumor inhibition (photodegradability)	[66]
RGD-PEG-Au@SiNRs	10 mg kg^−1^ /8 min with an interval 30 s	Tumor-targeted multimodal imaging-guided photothermal therapy	Tumor treatment (high photothermal conversion performance and good photothermal stability)	[63]
{(Au0)25-G5.NHAc-(PEG)14-(Fluo-4)2} DENPs	2.5 μM for 5 min	Tracking T cells via dual-modal CT and fluorescence imaging	T cell monitoring and tracking (water solubility, high x-ray attenuation coefficient, and good cytocompatibility)	[72]
PEG-PTyr(^125^I)-ICG PMs	100 μCi for 5 min	Noninvasive multimodality imaging-guided precision PTT	Clinical tumor theranostics (biocompatibility, size/photo/radiolabel stability, passive tumor-targeting ability)	[83]
mPEG&cRGD-g-PAsp@MnO	0.5 mg per kg at echo time 15 ms	MRI	T1-weighted MRI diagnosis of tumor (biocompatibility and stability)	[152]
PEG coated IONPs	0.2 mg ml^−1^	Magnetic resonance molecular imaging	Solid tumor tracking and monitoring	[153]
Sialic acid (SA)-modified SPIO-loaded mesoporous polydopamine (SAPEG-MPDA@SPIO NPs)	50 μg/ml	MRI-guided near-infrared photothermal therapy	Good water solubility exhibited good stability and outstanding photothermal conversion efficiency (35.4%), and produced superior magnetic resonance-enhanced imaging	[154]

## Green Nanotechnology in Multimodal Imaging and Therapy

Scientific advancements are ultimately driven by the goal of enhancing human health and overall well-being. While conventional therapeutic approaches have made significant strides, there is an increasing need for innovative methods that ensure both safety and efficacy in medical treatments. One such growing field is green nanotechnology, a sub-discipline of green technology that integrates the principles of green engineering and green chemistry. Green nanotechnology plays a pivotal role in environmental sustainability through the development of nanomaterials and nanoproducts, particularly in the form of phytoformulations [84]. These innovations not only reduce environmental impact but also prioritize the health and safety of both humans and ecosystems.

As such, green nanotechnology represents a promising approach to achieve sustainable development while addressing critical global health and environmental challenges. The use of plants in NP synthesis is driven by their widespread availability and the rich diversity of metabolites they contain, such as vitamins, antioxidants, and nucleotides [85]. This biological diversity makes plants an ideal resource for NP production. For example, AuNPs have garnered significant interest due to their tunable size, shape, and surface characteristics. Similarly, various copper (Cu) and copper oxide (CuO) NPs have been successfully synthesized using plant extracts. Other important metal oxide NPs, such as titanium dioxide (TiO_2_) and zinc oxide (ZnO), have also been produced from plant sources, further highlighting the potential of green nanotechnology in advancing sustainable development while minimizing environmental and health risks [16,86].

### Green synthesis of NPs for imaging

“Green synthesis” or “green approaches” refer to the eco-friendly production of metal NPs using plants, plant extracts, flowers, and leaves as an alternative to conventional chemical and physical methods. By combining different organic and inorganic materials, researchers can synthesize hybrid NPs that exhibit unique properties derived from each constituent material, leading to the formation of nanostructures with novel characteristics [87]. Plants, algae, bacteria, yeast, fungi, and other organisms have been utilized in the synthesis of these hybrid NPs. These materials have significant importance in the biomedical field, especially as nanocarriers for drug delivery, tissue engineering frameworks, imaging, etc. Additionally, green synthesis offers several advantages over traditional chemical and physical methods. These methods are nontoxic, avoids environmental pollution, and is generally more eco-friendly, cost-effective, and sustainable. These benefits make it an attractive alternative, especially for applications aiming to reduce environmental impact. However, there are still some limitations including that sourcing of suitable raw materials can be difficult due to limited availability and seasonal constraints. Additionally, the synthesis process often takes longer, and achieving consistent particle size and shape in the final product can be difficult. The synthesis process often demands high energy, extended reaction times, and additional chemical reagents, challenging the “green” label [88,89]. NPs produced through this greener method typically have a uniform chemical composition and exhibit minimal defects. The utilization of extracts from plants and microorganisms in the biological synthesis of NPs has gained widespread interest for its potential in sustainable nanotechnology.

One of the NPs that have been synthesized through plant-based components is AgNPs. This green synthesis process involves a Ag ion solution and a biological reducing agent. The most efficient and cost-effective approach to producing AgNPs relies on the reduction and stabilization of Ag ions through biological molecules including amino acids, proteins, polysaccharides, vitamins, alkaloids, phenolics, terpenes, and saponins. Nearly all plant species can facilitate the synthesis of AgNPs. Similarly, AuNPs have gained significant attention due to their ease of synthesis, surface functionalization, and distinct properties, including high medicinal potential, low toxicity, and exceptional biocompatibility [90]. In biogenic synthesis, various chemical constituents act as reducing agents to convert Au ions into NPs. Studies suggest that biomolecules like flavonoids, phenols, and proteins play a key part in the reduction of Au ions and subsequent capping of AuNPs in extracts of plants [91].

Zinc oxide NPs (ZnONPs) have also been studied in past years because of their diverse usage in biomedicine, cosmetics, optics, and electronics. Numerous studies have explored the plant-based generation of ZnONPs, highlighting their cost-effectiveness, safety, and simplicity. These NPs can be synthesized from various parts of plants, such as flowers, seeds, roots, and leaves. ZnONPs possess a high exciton binding energy of 60 meV and a wide bandgap of 3.37 eV, contributing to their remarkable semiconductor properties. Copper NPs (CuNPs), synthesized via the reduction of aqueous Cu ions using different plant extracts, offer a cost-effective alternative to AuNPs and AgNPs. Their formation is typically confirmed by an ultraviolet (UV)–visible spectrometer peak at 578 nm. However, concerns regarding the biosafety of CuNPs remain. Additionally, other metals such as nickel (Ni) and manganese (Mn) have been explored for NP synthesis. Metals like titanium (Ti), palladium (Pd), cerium (Ce), and platinum (Pt) have also been utilized in the plant-based production of NPs for various biomedical and industrial applications [84].

#### Environmental impact of conventional NP synthesis versus green approaches

The environmental influence of traditional NP synthesis approaches, commonly used in image-guided therapy, is a growing concern due to the reliance on toxic solvents, high energy consumption, and hazardous byproducts. Conventional techniques, such as chemical reduction and sol–gel processes, often generate harmful waste and contribute to environmental pollution. Further, in these NP-based drug delivery methods, materials including metallic or synthetic polymers may pose long-term risks of toxicity to both patients and the surroundings. In contrast, green approaches to NP synthesis use eco-friendly solvents, plant extracts, and biocompatible reducing agents [92]. These green methods reduce the ecological footprint by minimizing waste production, energy use, and the release of toxic substances. Additionally, NPs synthesized through green approaches exhibit comparable, if not superior, biocompatibility and effectiveness in image-guided therapy, making them a promising path for both therapeutic efficacy and environmental sustainability. Targeted therapy using NPs improves drug efficacy by delivering therapeutic agents directly to specific cells, tissues, or disease sites, reducing off-target effects and minimizing the required dosage. Key strategies for achieving this targeted delivery include surface functionalization of NPs with ligands that bind to specific receptors overexpressed in diseased tissues, such as cancer cells. These ligands may include antibodies, peptides, or aptamers, which enable NPs to selectively interact with their target, improving therapeutic outcomes and minimizing side effects [93,94].

#### Applications of green nanotechnology in image-guided therapy

**Fig. 5.**
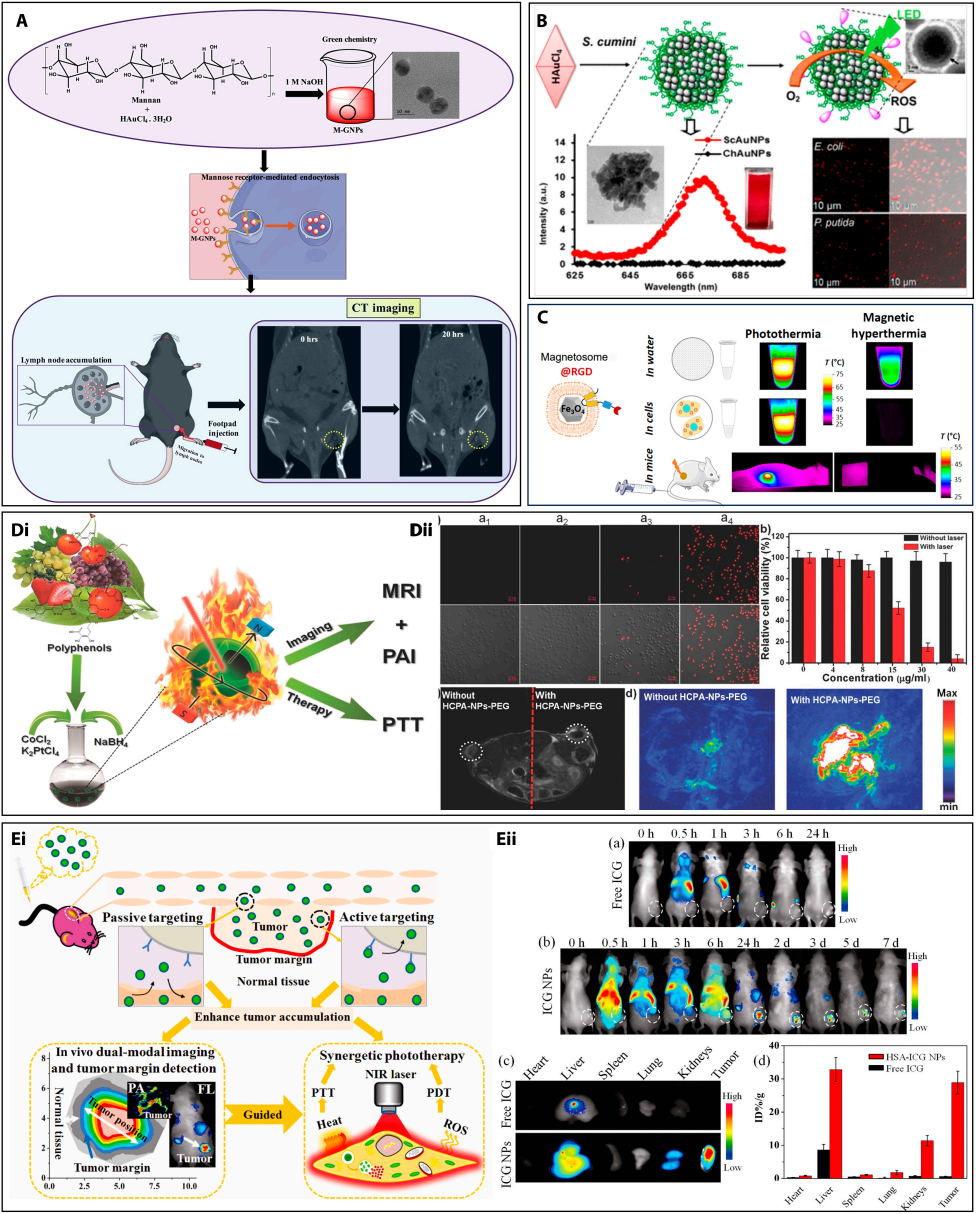
(A) Schematic illustration of the green chemistry synthesis of M-GNPs for CT imaging (reprinted with copyright permission from [97] Elsevier). (B) Synthesis of Au-based phototheranostic nanoagents from plant extracts for photodynamic therapy [reprinted with permission from [98] with copyright (2017) American Chemical Society]. (C) Targeted thermal therapy utilizing genetically engineered RGD-tagged magnetite magnetosomes (reprinted from [102] with copyright permission obtained from Elsevier). (D) (i) Schematic depiction of the synthesis and theranostic applications of HCPA-NPs. (ii) CLSM (confocal laser scanning microscopy) images of HepG2 cells stained with propidium iodide (PI) show dead cells in the top panels and the overlay with bright-field images in the bottom panels, under various treatments: (a_1_) 8-min laser, (a_2_) HCPA-NPs-PEG only, (a_3_) HCPA-NPs-PEG with 2-min laser, and (a_4_) HCPA-NPs-PEG with 8-min laser. Cell viability data for HepG2 cells exposed to different concentrations of HCPA-NPs-PEG, with and without laser irradiation, are also presented. T2-weighted MRI scans of mice display tumors without (left) and with (right) HCPA-NPs-PEG, marked by white circles. Additionally, photoacoustic (PA) images of the tumor site are shown for conditions with and without HCPA-NPs-PEG injection (reprinted from [103] with copyright permission obtained from Wiley). (E) (i) Smart HSA-ICG NPs synthesized by the assembly for dual-modal imaging-guided synergistic phototherapy in cancer treatment. (ii) PDT and PTT images after treating with the biocompatible NPs in tumor-induced mice model [reprinted with permission from [104] with copyright (2014) American Chemical Society].

Green nanotechnology has become a promising approach to mitigate the toxicity of nanomaterials in imaging by using eco-friendly and cost-efficient methods that utilize living organisms and biomolecules [95]. Fazal et al. [96] reported the first green-synthesized anisotropic and cytocompatible AuNPs without the use of capping agents for photothermal therapy. These AuNPs demonstrated excellent x-ray contrast during CT testing, confirming their potential as a contrast agent. Mannan-capped gold NPs (M-GNPs) were biologically targeted as contrast agents for x-ray CT imaging, where mannan acted as a reducing and stabilizing agent, which allowed M-GNP uptake by antigen-presenting cells via mannose receptors. The NPs, averaging 9.18 nm in size, showed concentration-dependent x-ray attenuation with a maximum Hounsfield unit (HU) of 303.2. Local administration enhanced contrast in popliteal lymph nodes, demonstrating the potential of M-GNPs for targeted CT imaging (Fig. 5A) [97]. Similarly, *Syzygium cumini* fruit extract was used to synthesize fluorescent AuNPs with antioxidant potential. These Au-based phototheranostic nanoagents (PTNAs) were functionalized with photosensitizers or imaging agents, exhibiting fluorescence, singlet oxygen generation, and antimicrobial activity under green light-emitting diode (LED) light. Comparisons with chitosan-based PTNAs confirmed their potential for photodynamic therapy in cancer and microbial infections, as characterized by confocal laser scanning microscopy (Fig. 5B) [98]. While both mannan and plant extract-based synthesis methods are green and biocompatible, M-GNPs demonstrated superior CT contrast (HU of 303.2) and cell targeting via receptor-mediated uptake, whereas *S. cumini*-derived PTNAs excelled in photodynamic and antimicrobial applications under LED illumination. Thus, the selection of plant extract or biological material significantly impacts the functional properties of the resulting NPs.

In the case of brain tumor targeting, IONPs and SPIONs are commonly used, although they often require polymeric or hydrophilic coatings to reduce cytotoxicity and enhance drug/gene loading [99,100]. Chemically synthesized ferro/ferrimagnetic NPs, despite their popularity in brain cancer theranostics, can exhibit low targeting efficiency and poor internalization, reducing radio-enhancement efficacy. Magnetosomes, produced by magnetotactic bacteria (MTB), offer a promising alternative. These iron-rich magnetic NPs, encapsulated in a lipid bilayer, are biocompatible without requiring further functionalization and can be efficiently used [100]. Boucher et al. [101] demonstrated the first proof of concept for using magnetosomes as molecular imaging probes for MRI. They developed a rapid, one-step production method for RGD-tagged magnetosomes, which showed significant internalization into U87 tumor cells during in vitro studies (Fig. 5C).

In glioblastoma-bearing mice, intravenous injection of these magnetosomes led to rapid tumor accumulation due to enhanced EPR (electron paramagnetic resonance) effects. MRI scans taken 24 h post-injection revealed specific tumor contrast enhancement, which was further corroborated by histological data. This study confirmed the successful bio-integrated production of MRI molecular imaging probes using magnetosomes. Additionally, RGD-tagged magnetosomes have also been a promising platform for multivalent thermal cancer therapy. Comparative studies on the efficacy of photothermal and magnetic hyperthermia treatments demonstrated that photothermal therapy exhibited good performance, while magnetic hyperthermia was nearly completely suppressed by the cellular microenvironment [102]. In comparison to synthetic SPIONs, magnetosomes offer higher biocompatibility, require no additional surface modifications, and show enhanced tumor-specific contrast and uptake, indicating a clear advantage for use in MRI-guided cancer therapy.

Theranostic nanomedicines, which combine diagnostic and therapeutic functions in a single platform, are progressively significant in cancer treatment. A novel green synthesis method for hollow CoPt alloy NPs (HCPA-NPs) using plant polyphenols as templating agents was reported. This method allows size-controlled synthesis by adjusting polyphenol molecular sizes and is adaptable for producing other hollow alloy NPs with various metal compositions. HCPA-NPs exhibit good biocompatibility and are effective in magnetic resonance and photoacoustic dual-modal imaging, guiding photothermal therapy. This approach offers new avenues for the green synthesis of hollow NPs for theranostic applications (Fig. 5D) [103]. Similarly, another study developed human serum albumin-indocyanine green NPs (HSA-ICG NPs) through disulfide conjugations to enhance phototherapy for cancer treatment. HSA-ICG NPs exhibited high tumor accumulation (tumor-to-normal tissue ratio of 36.12 ± 5.12 at 24 h) and long retention in 4T1 tumor-bearing mice. These NPs enabled precise imaging of tumors using NIR fluorescence and photoacoustic imaging, and induced ROS and hyperthermia under NIR laser irradiation for synergistic PDT/PTT. After treatment, tumors were completely suppressed without recurrence or toxicity, indicating HSA-ICG NPs as a promising theranostic platform for imaging-guided cancer phototherapy (Fig. 5E) [104]. Notably, both HCPA-NPs and HSA-ICG NPs influences green synthetic strategies and exhibit high imaging resolution and therapeutic efficacy. However, HSA-ICG NPs showed high tumor-to-normal tissue ratio and complete tumor suppression, which demonstrated their good in vivo performance in cancer phototherapy. Green synthesis methods using biological agents such as plant extracts, bacterial products, or serum proteins yield diverse NPs with specific advantages. While AuNPs and magnetosome-based NPs is good in imaging and cellular targeting, albumin-based NPs demonstrate excellent tumor selectivity and therapeutic outcomes.

## AI in Medical Imaging, Data Analysis, and Personalized Health

**Fig. 6.**
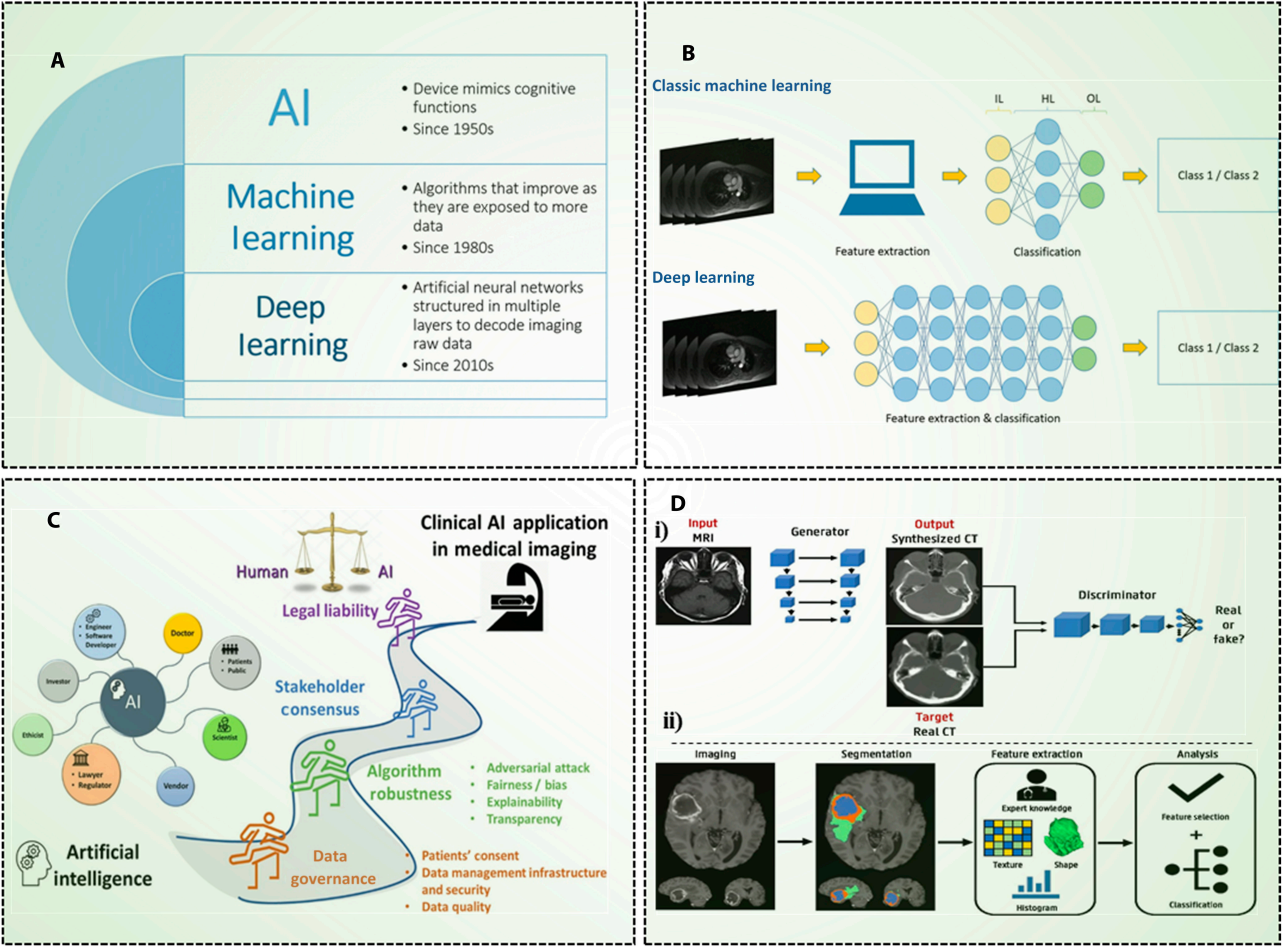
Overview of AI in medical imaging. (A) Illustration of the difference between AI, ML, and DL. (B) Classical ML and DL show neural networks structured in distinct layers (reprinted from [146] under Creative Commons Attribution 4.0 International License). (C) Obstacles to be overcome in the use of AI in clinical medical imaging, from patient permission to algorithm robustness [reprinted from [147] with copyright (2022) Elsevier]. (D) (i) Example of image synthesis of CT from MRI by using GAN. (ii) A typical workflow in radionics consists of segmentation using segmentation algorithms (reprinted from [148] under Creative Commons Attribution 4.0 International License).

AI is a field of computer science that involves the development of algorithms and systems capable of analyzing complex data and performing tasks that typically require human intelligence [105]. Within AI, ML refers to algorithms that learn patterns from data to make decisions or predictions. A key type of ML is the artificial neural network (ANN), inspired by the human brain’s structure. When ANNs are composed of multiple interconnected layers, they are referred to as deep neural networks (DNNs)—the core of DL (Fig. 6A and B).

AI technology has revolutionized the field of medical imaging, allowing for more accurate diagnoses of diseases and abnormalities, validation of contrasting agents for in vivo applications, streamlined workflows, and improved patient outcomes by personalized medicine [106] (Fig. 6C). Their potential is evident in various applications, including risk modeling and stratification, personalized screening, diagnostic tasks (such as identifying molecular disease subtypes), predicting therapeutic responses, and forecasting patient outcomes [107]. AI applications in medical imaging can be developed using 2 distinct architectures, each with its own typical workflow: (a) traditional ML, which relies on manually designed features, such as radiomic features extracted from segmented images, and (b) DL, which enables end-to-end learning from raw images by automatically extracting deep features [108]. ML models can classify patterns in imaging datasets, while DNNs are particularly powerful in tasks such as tumor detection, organ segmentation, and multimodal image fusion due to their ability to learn hierarchical representations of imaging data (Fig. 6Dii).

A necessary first step in any feature segmentation process is precisely identifying the region of interest (ROI) within the sample [109]. Liver and liver tumor, brain and brain tumor, optic disc, cell, lung, pulmonary nodules, and cardiac image segmentation are common medical image segmentation concerns [110]. A popular encoder–decoder network for semantic medical picture segmentation is called UNet [111]. Nevertheless, this approach is unable to properly learn distant semantic knowledge and global information. A brand-new network known as the O-Net, which completely utilizes both global and local information to improve medical picture segmentation and classification by combining the benefits of CNN (convolutional neural network) and transformer, demonstrates strong generalization skills and competitive performance in the segmentation and classification tests [112]. In a similar vein, a novel U-Net-based medical picture segmentation architecture called R2U++ aims to bridge the semantic divide between the encoder and the decoder. A set of outputs from different depths enhances the performance of foreground items that appear in the images at different scales. The updated architecture includes multi-depth models embedded in it [113]. To extract more precise semantic feature information from medical pictures, attention-guided cross-deconvolution networks (DCACNet) and dual context aggregation were utilized [114]. UNeXt is an image segmentation network based on convolutional multilayer perceptrons (MLPs). For the automatic segmentation of medical pictures, a multipath feature fusion CNN (MF2-Net) with innovative and effective spatial group convolution (SGC) modules has been reported [115].

Modern backbone models are surpassed by ResGANet in tasks involving the classification of medical images. Without altering the network architecture, applying it to various segmentation networks can enhance the baseline model in medical picture segmentation tasks [116]. Moreover, Cerberus, a multi-task learning technique, has been used to segment and classify nuclei, glands, lumina, and other tissue regions by utilizing data from numerous independent data sources [117]. AI is still limited in its ability to classify and segment images, despite its impressive progress in these areas. Due to the strong correlation between the caliber, variety of data, and model performance, one difficulty is the requirement for large labeled datasets for training. The lack of transparency in DL models makes it difficult to comprehend their decision-making processes, which leads to interpretability issues. Developments in unsupervised learning, transfer learning, and the creation of more interpretable AI models are some ways that future perspectives plan to tackle these issues [118].

The DL approach outperformed the other methods in terms of its capacity to learn a nonlinear mapping from one image domain to another. DL techniques fall into 2 categories: supervised and unsupervised. Model training in supervised DL techniques needed paired pictures. For the neural network model to learn an end-to-end mapping in the MR/CT synthesis job, MR and CT images must first be well-registered. These pictures are then used as inputs and associated labels [119]. The accuracy of learning-based supervised and unsupervised image synthesis techniques for pseudo-MR/CT-generating tasks was investigated in a study. Two exemplary U-Net and CycleGAN networks were presented as examples of supervised and unsupervised learning techniques. Supervised methods are more accurate than unsupervised ones [120]. In several medical image synthesis applications, generative adversarial network (GAN) models with CNN backbones have emerged as state-of-the-art recently (Fig. 6Di).

Medical image synthesis has been the subject of numerous studies conducted recently. A new GAN method for medical image synthesis called ResViT was developed in a different study. It makes use of the contextual sensitivity of vision transformers, the accuracy of convolution operators, and the realism of adversarial learning. The synthesis of missing sequences in CT images and multi-contrast MRI from MRI can be performed by using ResViT. In terms of quantitative metrics and qualitative observations, ResViT has many advantages over rival transformer and CNN-based techniques [121]. It has been demonstrated to develop 3-dimensional (3D) high-resolution images using a hierarchical GAN model. Trials using brain MRI and 3D thorax CT demonstrate that hierarchical amortized generative adversarial networks (HA-GAN) performs at the cutting edge of image synthesis and therapeutic applications [122]. Multimodality-guided synergistic neural network (MMgSN-Net) synthesized virtual contrast-enhanced MRI for patients with nasopharyngeal cancer. Our MMgSN-Net performed better than U-Net, CycleGAN, and Hi-Net, according to the results of the quantitative assessments [123]. Generative AI, such as the StyleGAN-based StylEx framework, offers a novel approach to enhance interpretability in medical imaging for precision healthcare. By generating counterfactual visualizations, StylEx identifies fine-grained visual attributes relevant to classification tasks across modalities like fundus photos and chest x-rays. These visual cues, evaluated by interdisciplinary experts, reveal both clinically recognized patterns and novel associations influenced by socio-structural factors. This approach enables hypothesis generation, supports bias detection, and improves model transparency [124]. GANs have been effectively utilized to generate missing imaging sequences, such as FLAIR or T1-weighted MRI, to support tumor segmentation models, while diffusion models offer higher-resolution reconstructions with reduced artifacts. Furthermore, multimodal generative AI, integrating visual data with clinical text via vision-language modeling, holds promise for automated report generation and cross-modality translation. By producing realistic and diverse synthetic data, generative AI reduces dependency on large annotated datasets, making it an ideal complement to sustainable nanotechnology strategies in image-guided therapy, where minimizing patient exposure to radiation or contrast agents is crucial [125]. A recent meta-analysis of 83 studies revealed an overall diagnostic accuracy of 52.1% for generative AI models, with performance varying by model and specialty. Although these systems remain inferior to expert physicians, their efficiency in processing vast clinical data makes them valuable for supporting decision-making, especially in resource-limited settings [126]. These findings underscore the evolving role of AI in healthcare and highlight the need for transparency, rigorous validation, and contextual understanding of AI limitations to ensure safe and effective integration in clinical decision-making and image-guided therapeutic strategies.

Large visual models (VLMs) have undergone significant research-driven evolution in medical image analysis, transitioning from simple modality fusion approaches to sophisticated, general-purpose multimodal large models. Initial efforts focused on aligning visual and textual features using independent encoders and basic fusion strategies. Progressively, the adoption of visual-semantic embeddings and attention mechanisms improved cross-modal interactions. The introduction of transformer-based architectures, such as ViLBERT and CLIP, marked a turning point, enabling large-scale pretraining with contrastive and masked generation techniques. These advances facilitated robust zero shot and few shot learning, particularly valuable in medical domains with limited labeled data. Recent models demonstrate capabilities in fine-grained image text alignment, disease classification, segmentation, report generation, and cross-modality image synthesis. Nonetheless, despite these advances, critical limitations persist, including high computational costs, domain mismatch between pretraining and clinical data, and interpretability concerns [127].

### Integration of AI with nanotechnology for enhanced precision

The integration of nanotechnology and AI offers promising advancements in image-guided therapy. AI algorithms can be used to optimize the design and functionalization of NPs for targeted imaging and therapy. In turn, NPs enhance imaging quality and generate high-resolution data that improve the training and accuracy of AI models. This bidirectional interaction enables more precise diagnosis, real-time image interpretation, and personalized treatment planning. The combination of AI with nano-assisted imaging represents a significant advancement in medical technology, offering new possibilities for noninvasive, accurate, and efficient healthcare solutions. Nanomaterials enable controlled drug release by aligning dosing with specific pharmacokinetic and pharmacodynamic profiles, utilizing external stimuli like magnetic fields, electric fields, and US. A study demonstrated that charged AuNPs showed promise as CT contrast agents for enhancing image quality in small hepatocellular carcinoma (sHCC), enabling accurate tumor segmentation using AI models like 3D U-Net and Trans U-Net, and potentially overcoming key barriers in adaptive radiotherapy (ART) [128]. For instance, a ferrogel with Fe_3_O_4_ NPs can be manipulated magnetically to release drugs or even cells. To enhance personalized dosing, these systems should be integrated with real-time sensors to monitor drug levels, akin to insulin pumps. AI can further refine treatment by predicting drug–response relationships and tailoring therapy, as demonstrated by neural networks in radiotherapy and pharmacogenetic predictors in cancer treatment [129].

Implementing these technologies in nanomedicine could revolutionize clinical outcomes [130]. In a study, they applied the K-means++ algorithm to segment regions of interest (ROI) in MPI data from in vitro, in vivo, and ex vivo models, focusing on islet transplantation in mice. The algorithm effectively minimized noise and provided accurate segmentation, with linear correlations observed between islet numbers and total iron values (TIVs). This was consistent across all data types. Statistical validation via intraclass correlation coefficient demonstrated the model’s high performance, establishing K-means++ as a reliable method for MPI data segmentation and quantification in cell-based therapies [130]. In another study, targeting scavenger receptor-AI (SR-AI), which is prominently expressed by macrophages in atherosclerotic plaques, offers a promising approach for molecular imaging.

By conjugating ultrasmall superparamagnetic iron oxide particles (USPIO) with a specific SR-AI ligand, researchers demonstrated enhanced iron uptake by macrophages in vitro and increased plaque accumulation in vivo compared to nontargeted USPIO. This targeted imaging approach led to significantly improved contrast in atherosclerotic plaques, as evidenced in both murine and humanized models. These findings suggest that SR-AI-targeted USPIO-based contrast agents could be highly effective for the in situ detection of inflammatory atherosclerotic plaques. The integration of AI algorithms, particularly DL, with USPIO-enhanced imaging facilitates automated detection, classification, and quantification of inflammatory lesions. This synergy improves diagnostic accuracy, contrast-to-noise analysis, and risk stratification. Consequently, USPIO-AI-based imaging holds significant potential for noninvasive, real-time assessment of disease activity, supporting precision diagnostics and personalized therapeutic strategies in cardiovascular and inflammatory disorders [131]. Further, AI algorithms can detect patterns, identify abnormalities, and assist in the interpretation of medical images with remarkable precision. Moreover, AI-powered imaging systems are capable of autonomously performing routine tasks, freeing up radiologists’ time to focus on complex cases. As the capabilities of AI continue to advance, its integration into imaging practices holds immense potential for enhancing healthcare delivery and transforming the field of diagnostics [132]. Applying AI to integrate data from many imaging modalities is leading to a holistic diagnostic method.

A study was conducted to assess AI’s ability to detect lung nodules on chest CT scans in patients with complex lung disease. AI was found to be 27 s faster on average and identified 8.4% of nodules that were missed by the radiologist. It achieved a sensitivity of 67.7% overall and correctly classified patients with 96.1% sensitivity. Compared to radiology reports, AI demonstrated 89.4% sensitivity and 82.5% specificity in the control group. AI significantly reduced assessment time per case and increased diagnostic confidence, showing promise for improving efficiency and accuracy in clinical practice [133]. Similarly, a study developed and validated the gastrointestinal artificial intelligence diagnostic system (GRAIDS) to detect upper gastrointestinal cancers using endoscopy imaging data from multiple hospitals in China. GRAIDS achieved high diagnostic accuracy with an average of 0.955 in internal validation and sensitivity comparable to expert endoscopists (0.942). It outperformed less experienced endoscopists (0.858 for competent, 0.722 for trainee) and showed strong positive (0.814) and negative (0.978) predictive values [134].

Integrating AI with NP-based imaging diagnostics involves several scientific challenges. Combining data from MRI, PET, and genomic sources enhanced by NPs requires AI systems that can handle complex and varied information. However, current AI models often struggle with this type of high-dimensional data. Tracking how NPs move and accumulate in tissues for accurate imaging also needs AI algorithms that are transparent and reliable, but most DL models do not clearly explain their decisions, making them hard to trust in clinical settings. Differences in NP size, shape, and surface features affect image quality and make it difficult to train AI consistently. In addition, imaging data can be noisy or vary between machines, causing models to overfit and perform poorly on new data. Finally, current regulatory systems are not designed to evaluate AI combined with nanotechnology, slowing down approval for clinical use [135].

## Synergy of Green Nanotechnology and AI in Personalized Health

The incorporation of AI with nanotechnology in nanomedicine holds great potential to improve treatment and healthcare delivery. Nanotechnology has enabled the growth of advanced delivery systems for drugs, diagnostic tools, and therapeutic interventions with enhanced capabilities in imaging, targeting, and therapy [136]. However, maximizing the safety and effectiveness of these nanomedical technologies requires personalized approaches to the unique characteristics and disease profiles of individual patients. AI-driven techniques facilitate precision medicine by classifying patient populations based on prognoses, treatment responses, and disease subtypes through the analysis of large datasets, such as genomic, proteomic, and medical imaging information [137]. Further, ML algorithms can recognize biomarkers related to disease progression and efficacy of the drug, allowing for the selection of optimal treatment strategies for individual patients. Furthermore, AI-powered nanomedicine systems enable real-time evaluation of drug delivery, pharmacokinetics, and treatment responses, supporting adaptable treatment plans and improving patient outcomes. Several case studies highlight the synergistic benefits of integrating AI with green nanotechnology in clinical settings [138]. These applications have shown promise in improving patient outcomes, reducing environmental impact, and advancing the development of sustainable medical practices.

## Conclusion and Future Perspectives

One of the key importance for advancing the use of NPs in medical imaging lies in improving the biocompatibility and bioavailability of nanomaterials. This ensures their safe and effective use in biological systems, minimizing potential toxicity while maximizing their functional benefits [139]. Nanomaterial-related challenges in multimodal imaging, such as toxicity, limited biocompatibility, poor stability, and environmental impact, hinder their broader use in medical applications. Traditional NPs can accumulate in tissues, causing adverse effects, and are often difficult to clear from the body. Green nanotechnology addresses these issues by using biodegradable, biocompatible materials derived from natural sources, reducing toxicity and improving safety. Additionally, green synthesis methods are eco-friendly, using fewer harmful chemicals and energy-intensive processes. This approach enhances NP stability and targeting efficiency, making multimodal imaging safer, more precise, and environmentally sustainable, thus advancing both patient care and environmental protection.

**Fig. 7.**
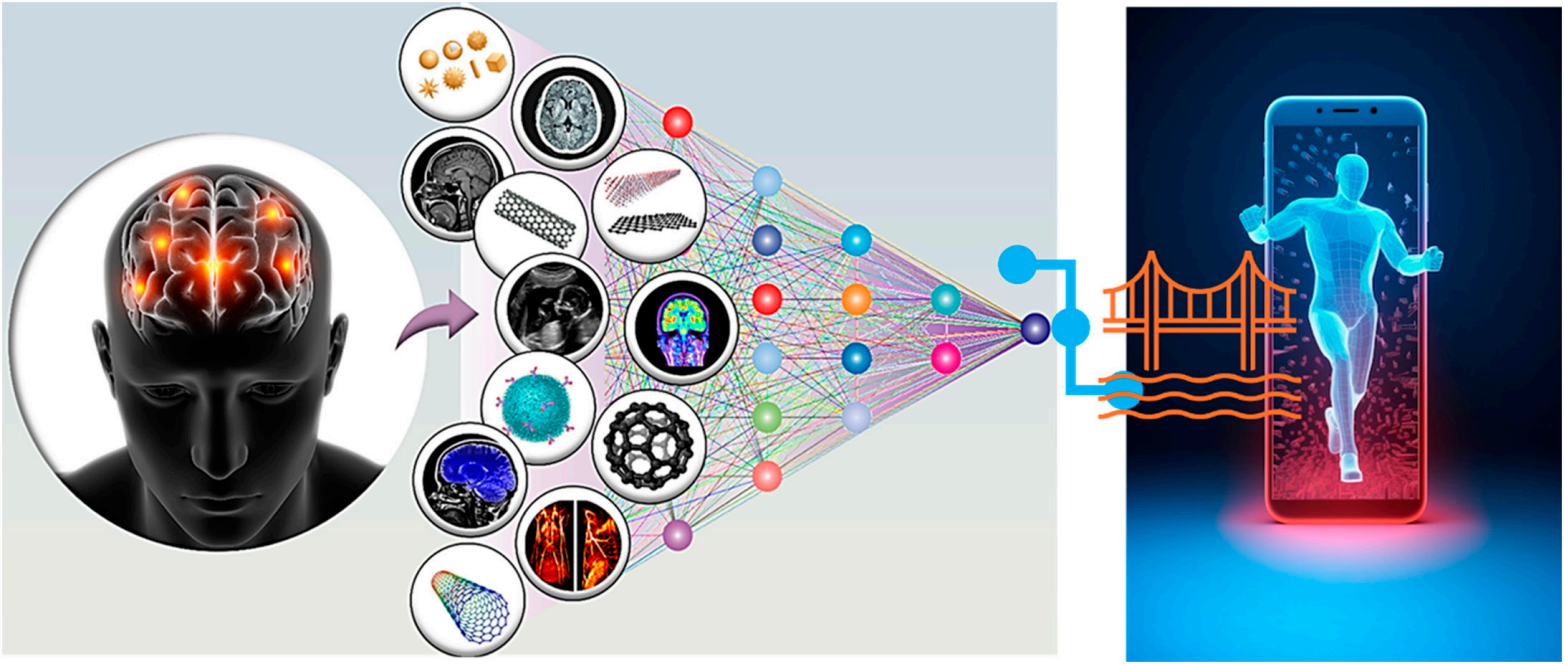
The convergence of AI, advanced medical imaging technologies, and sustainable nanomaterials in the realm of healthcare. The interaction of these technologies offers significant opportunities for personalized disease diagnosis, targeted therapies, and advancements in healthcare.

AI in medical imaging has demonstrated notable advancements, offering valuable tools for diagnostics and patient care. In the upcoming years, it is anticipated that general AI will perform better than humans in certain applications. The human–AI relationship may help humans become more intelligent and advance their status (Fig. 7) [132]. However, certain limitations persist. One significant issue that interferes with ML applications in the field of image analysis and prevents its growth is the lack of high-quality annotated medical imaging datasets [140]. The capacity of network models to learn image representations and process vast amounts of data efficiently using present computational approaches is the primary reason for the present development in the quality of medical image multiple-lesion detection. The majority of the existing multiple-lesion recognition algorithms for medical imaging are too complex and require a lot of repetitive annotations to suit the needs of medical applications [141].

Further, AI tools in image synthesis face limitations such as challenges in generating realistic high-resolution images and potential biases in training data. Hence, future research should focus on how to overcome these limitations, highlighting collaborative efforts for data access and quality improvement and generating models to enhance image quality, addressing ethical concerns, and integrating AI with human creativity for collaborative and diverse visual content creation. Moreover, efforts to prevent biases and promote fairness in AI applications are crucial, alongside the establishment of clear regulatory frameworks. Therefore, overcoming this limitation and combining NPs and multimodal imaging, along with AI integration, can improve their clinical applications. Nevertheless, continued research, standardization, and ethical considerations will be pivotal in harnessing the full potential of these innovations. By addressing these challenges, the field can move closer to realizing the full benefits of nanotechnology-enhanced medical imaging, marking a new era in precision medicine.
